# Association of LncRNA MEG3 polymorphisms with efficacy of neoadjuvant chemotherapy in breast cancer

**DOI:** 10.1186/s12885-019-6077-3

**Published:** 2019-09-05

**Authors:** Battseren Bayarmaa, Ziping Wu, Jing Peng, Yan Wang, Shuguang Xu, Tingting Yan, Wenjin Yin, Jinsong Lu, Liheng Zhou

**Affiliations:** Department of Breast Surgery, Renji Hospital, School of Medicine, Shanghai Jiaotong University, 160 Pujian Road, Shanghai, 200127 People’s Republic of China

**Keywords:** Breast cancer, MEG3 non-coding RNA, Neoadjuvant therapy, Cisplatin, Paclitaxel

## Abstract

**Background:**

Breast cancer is the most common malignancy in women, and neoadjuvant chemotherapy has been recommended to the patients with locally advanced breast cancer as the initial treatments. Long non-coding RNA (lncRNA) MEG3, an identified tumor suppressor, has been implicated in the development of various cancers. However, there is no data to evaluate the effect of MEG3 polymorphisms on neoadjuvant treatment in the breast cancer.

**Methods:**

Genotyping was performed using Nanodispenser Spectro CHIP chip spotting and Mass ARRAY Compact System. Univariate and multivariate logistic regression analyses were used to analyze the associations between the MEG3 polymorphisms and the pathological complete response (pCR). The disease-free survival (DFS) was estimated by the Kaplan-Meier method, and multivariate Cox proportional hazards models were used to calculate the hazard ratios (HRs) with a 95% confidential interval (CI).

**Results:**

A total of 144 patients with available pretreatment blood species were enrolled in the SHPD002 clinic trial of neoadjuvant chemotherapy for breast cancer. MEG3 rs10132552 were significantly associated with good response (Adjusted OR = 2.79, 95% CI 1.096–7.103, *p* = 0.031) in dominant model. Median follow-up time was 20 months. In multiple regression analysis, rs10132552 TC + CC (adjusted HR = 0.127, 95% CI 0.22–0.728, *p* = 0.02) and rs941576 AG + GG (adjusted HR = 0.183, 95% CI 0.041–0.807, *p* = 0.025) were significantly associated with good DFS. MEG3 rs7158663 (OR = 0.377, 95% CI 0.155–0.917, *p* = 0.032) were associated with a low risk of hemoglobin decrease in dominant models.

**Conclusions:**

LncRNA MEG3 polymorphisms were associated with the chemotherapy response and toxicity of paclitaxel and cisplatin. The result indicates that MEG3 polymorphisms can be considered as the predictive and prognostic markers for the breast cancer patients.

**Trial registration:**

Retrospectively registered (ClinicalTrials. Gov identifier: NCT02221999); date of registration: Aug 20th, 2014.

**Electronic supplementary material:**

The online version of this article (10.1186/s12885-019-6077-3) contains supplementary material, which is available to authorized users.

## Background

Long non-coding RNAs constitute a heterogeneous group of the ncRNAs that are longer than 200 nucleotides. LncRNAs can regulate gene expression at epigenetic, transcriptional, and post-transcriptional levels, and can affect drug response and toxicity in cancer patients [1]. It was reported that some LncRNAs were tumor suppressor in breast cancer, such as growth arrest-specific 5, neuroblastoma associated transcript 1, and maternally expressed 3 (MEG3) [2]. We also found that MEG3 was downregulated in the ER positive breast cancer in our previous study [3]. MEG3 is chromosomally located at 14q32.3 in humans [4]. In a pooled analysis, a low expression of MEG3 showed to be associated with low overall survival in cancer patients, but not in the breast cancer patients [5]. However, single nucleotide polymorphisms (SNPs) in MEG3 were reported to affect cell phenotypes and cause the risk of developing cancer [6] and the chemotherapy toxicity [7] in other cancers. There have been no analyses published to date of association between MEG3 and chemotherapy response in breast cancer patients.

Neoadjuvant chemotherapy has been recommended to the patients with locally advanced breast cancer as the initial treatments. Many clinical trials, such as NSABP B18 and B27, have confirmed that patients with neoadjuvant chemotherapy achieved pCR could be a surrogate for their prognosis [8, 9]. Hormone receptor status and human epidermal growth factor receptor − 2 (HER2) expression were long known as predictors for chemotherapy response [10, 11]. The addition of platinum to a neoadjuvant chemotherapy in some subtype breast cancer could increase the proportion of patients achieving a pCR [12, 13]. Platinum containing chemotherapy was recommended as a preferred regimen for recurrent or stage IV patients with triple-negative tumors and germline BRCA1/2 mutation in 2019 NCCN clinical practice Guidelines [14]. However, rate of pCR still differs between the subset of patients with same biologic phenotype. We need to look for new markers to predict response independent from the established biological markers. The above data prompted us to conduct this prospective-retrospective analysis of the MEG3 lncRNA polymorphisms in available pretreatment blood specimens of patients enrolled in a clinic trial of neoadjuvant chemotherapy. The efficacy of paclitaxel and cisplatin as neoadjuvant setting has been studied in the SHPD001 trial [15], and the SHPD002 trial, which randomized to combine chemotherapy with endocrine therapy or not, will further prospectively estimate the efficacy of the regimen. Our prespecified objective was to determine whether the certain lncRNA polymorphisms could be the biomarkers to predict the benefit or prognosis. Here we hypothesized that these lncRNA polymorphisms would play important role in response to chemotherapy in breast cancer.

## Methods

### Study subjects

Consecutive, breast cancer patients were collected as part of a clinical trials SHPD002 for patients with locally advanced breast cancer (ClinicalTrials. Gov identifier: NCT02221999). One hundred and forty-four patients with information of SNPs were identified for analysis. The blood samples were collected between September 2015 and August 2017. Women aged ≥18 years old with histologically confirmed locally advanced invasive breast cancer were included. For all patients, paclitaxel 80 mg/m^2^ was given weekly on day 1 for 16 weeks, and cisplatin 25 mg/m^2^ was given on days 1, 8 and 15 every 28 days for 4 cycles. Patients with hormone receptor-positive cancer or premenopausal patients with triple negative breast cancer were randomized to concurrently receive endocrine therapy or not. Endocrine therapy included letrozole for postmenopausal women and gonadotropin releasing hormone agonist for premenopausal women (Additional file 1: Figure S1). HER2 positive patients could have trastuzumab concurrently with the chemotherapy in the neoadjuvant setting. The trastuzumab was given every week at 4 mg/kg (cycle1), followed by 2 mg/kg. In this explore analysis we used near pCR which was defined as only a few scattered tumor cells remained or that the residual tumor was < 0.5 cm in size [15, 16]. Tumor size and node status were assessed by combining physical examination with magnetic resonance imaging and ultrasound. ER, PR, Ki-67 and HER2 were performed on paraffin-embedded tumor samples from biopsy. Ki-67 levels were recorded as a continuous value, and a ki67 value of > 20% was high expression according to the Saint Gallen consensus [17]. DFS was defined as the time from surgery to local recurrence, original metastasis, second primary cancer or patient mortality. Informed consent was obtained from all individual participants included in the study.

### SNP selection and genotyping

Whole blood was collected before treatment and stored at − 80 °C. DNA extraction was performed using the TIANamp Genomic DNA Kit. A total of 3 potentially fictional SNPs of MEG3 LncRNA were selected in public database (NCBI/TargentScan), whose minor allele frequency > 0.1; located in the 3’UTR region or 5’UTR region and were reported to be susceptible factors or predictors in other tumors. MEG3 rs10132552, rs941576 and rs7158663 are the most studied lncRNAs involved in tumorigenesis and drug response. Genotyping was performed using Nanodispenser Spectro CHIP chip spotting and Mass ARRAY Compact System (Sequenom, San Diego, CA, USA) by Shanghai Benegene Biotechnology Co., LTD. Detailed primer sequences were provided in the Additional file 1: Table S1. Genotypes were determined with Typer software using default settings after auto clustering. Deidentified specimens were used to make sure that all assays were performed blinded to clinical outcome.

### Statistical analysis

Each SNP was explored in different comparison models in this analysis. For MEG3 rs10132552, genotype TT was used as reference; odds ratio (OR) for TC and CC were computed for additive model. Both TC and CC were combined and compared against TT as reference for dominant model. Recessive model (CC vs TC + TT) and co-dominant model (TT + CC vs TC) were also estimated. The Fisher exact east was used to test deviation form Hardy Weinberg Equilibrium and Chi-square tests were used to test the association of genotype with clinical characteristics. Multivariate logistical regression was conducted to calculate the association of each genotype with the efficacy and toxicities. Some regularly used clinical and biological characteristics were adjusted in the logistic and cox regression. The DFS was estimated by the Kaplan-Meier method, and multivariate Cox proportional hazards models were used to calculate hazard ratios (HRs) and 95% CIs. All statistical analyses were performed using PASW Statistics 18 software (IBM Co, Armonk, NY, USA). All tests were two-sided and *p* < 0.05 was considered significant.

## Results

### Patients clinical characteristics and genotype distribution

Among all the eligible patients, pretreatment blood species were available for 144. One hundred and twenty-one (84%) patients had hormonal receptor positive breast cancers. Fifty-two (36.1%) cancers were Her2 overexpressed. Fifteen (10.4%) patients had triple negative breast cancer. The near pCR rate was 37.5% for the entire cohort (Table 1). The genotype frequencies of all the SNPs were in Hardy-Weinberg equilibrium. MEG3 rs10132552 was significantly associated with tumor size in its recessive model (*p* = 0.022) and additive model (*p* = 0.007). Patients containing T allele in rs10132552 were likely to have larger or more invasive tumor (percentage of T3 and T4: TT 55.3% and TC 52.6%) compared with the CC genotypes (12.5%). Patients containing T allele in rs10132552 had higher level of ki67, while the proportions of high ki67 level in TT and TC genotype were 71.6 and 85.2%, which were significantly higher than that of CC genotype (50%) (Table 2). Polymorphisms in rs941576 and rs7158663 were not associated with clinical or biological characteristics (Additional file 1: Table S2).

**Table 1:** Baseline clinical characteristics of all patients

Characteristics	Number of Patients (n)	Percentage (%)
Age (years)		
≥ 50	58	40.3
< 50	86	59.7
Tumor stage		
T1–2	68	47.2
T3–4	73	50.7
Unknown	3	2.1
ER status		
Postive	102	70.8
Negative	42	29.2
PR status		
Positive	114	79.2
Negative	30	20.8
HER2 expression		
Positive	52	36.1
Negative	92	63.9
Ki67 status		
Low expression	33	22.9
High expression	103	71.5
unkown	8	5.6
Subtype		
Luminal A-like	12	8.3
Luminal B-like	108	75
HER2 positive (non lumninal)	9	6.3
Triple negative	15	10.4
Pathological response		
Complete response (include near pCR)	54	37.5
Partial response	83	57.6
Stable disease	7	4.9
Progression disease	0	0

**Table 2:** Association between MEG3 rs10132552 and clinic-pathological parameters of breast cancer patients

	rs10132552 n(%)			*P* value			
	TT	TC	CC	Dominant	Recessive	co-dominant	Additive
T stage							
1~2	34(44.7)	27(47.4)	7(87.5)	0.37	0.022*	0.867	0.07
3~4	42(55.3)	30(52.6)	1(12.5)				
Lymph node status							
Negative	8(11)	10(18.2)	2(25)	0.184	0.397	0.346	0.364
Positive	65(89)	45(81.8)	6(75)				
ER							
Negative	22(28.2)	18(31)	2(25)	0.783	0.79	0.686	0.905
Positive	56(71.8)	40(69)	6(75)				
PR							
Negative	15(19.2)	14(24.1)	1(12.5)	0.607	0.55	0.423	0.656
Positive	63(80.8)	44(75.9)	7(87.5)				
HER2							
Negative	54(69.2)	34(58.6)	4(50)	0.147	0.4	0.28	0.312
Positive	24(30.8)	24(41.4)	4(50)				
Ki67							
Low expression	21(28.4)	8(14.8)	4(50)	0.221	0.08	0.037*	0.045*
High expression	53(71.6)	46(85.2)	4(50)				
Menopausal status							
Premenopausal	35(44.9)	25(43.1)	2(25)	0.632	0.289	0.992	0.557
Postmenopausal	43(55.1)	33(56.9)	6(75)				

Abbreviations: *ER* estrogen receptor, *PR* progesterone receptor, *HER2* human epidermal growth factor receptor − 2

*P < 0.05

### LncRNA polymorphisms and response to chemotherapy

Patients with MEG3 rs10132552 were significantly associated with pCR in dominant model (TC + CC vs. TT OR = 2.396, 95% CI 1.202~4.777; *p* = 0.013) and in additive model (TC vs. TT OR = 2.376, 95% CI 1.164~4.847; *p* = 0.017). In another word, patients with TC + CC genotype had a significantly higher pCR rate compared with TT genotype (48.5% vs. 28.2, *p* = 0.012) (Table 3). The association is particularly seen in the hormone receptor positive patients(TC + CC vs. TT OR = 2.773, 95% CI 1.263~6.087; *p* = 0.011), but not in the hormone receptor negative patients (TC + CC vs. TT OR = 1.143, 95% CI 0.205~6.366; *p* = 0.879).

**Table 3:** Association between lncRNA MEG3 polymorphisms and pCR rate in different comparison models

SNP	Dominant model Genotypes	pCR n (%)	Non-pCR n (%)	P	Dominant model		Recessive model		Additive model		
					OR(95% CI)	*P*	OR(95% CI)	*P*	OR(95% CI)		*P*
rs10132552	TT	22(28.2)	56(71.8)	0.012*	2.396(1.202~4.777)	0.013*	1.72(0.412~7.181)	0.457	TC	2.376(1.164~4.847)	0.017*
	TC + CC	32(48.5)	34(51.5)						CC	2.545(0.585~11.082)	0.213
rs941576	AA	22(31.9)	47(68.1)	0.182	1.59(0.803~3.146)	0.183	2.194(0.563~8.554)	0.258	AG	1.479(0.731~2.994)	0.277
	AG + GG	32(42.7)	43(57.3)						GG	2.67(0.653~10.926)	0.172
rs7158663	GG	28(34.1)	54(65.9)	0.339	1.393(0.705~2.75)	0.34	2.959(0.678~12.916)	0.149	AG	1.227(0.602~2.503)	0.573
	AG + AA	26(41.9)	36(58.1)						AA	3.214(0.716~14.44)	0.128

Abbreviations: *pCR* pathological complete remission, *OR* odds ratio

*P < 0.05

The multivariate regression analysis demonstrated that MEG3 rs10132552 was statistically significant associated with good response (Adjusted OR = 2.79, 95% CI 1.096–7.103, *p* = 0.031) in dominant model. High ki67 level (Adjusted OR = 1.059, *p* < 0.001), HER2 overexpression (Adjusted OR = 11.718, *p* < 0.001) were also significantly associated with good efficacy. However, patients with old age (Adjusted OR = 0.951., *p* = 0.035) and positive hormonal receptors (Adjusted OR = 0.241, *p* = 0.022) were less likely to have good response (Table 4). Meanwhile, MEG3 rs941576 and rs7158663 polymorphisms were not associated with the response to chemotherapy in neither univariate nor multivariate analyses.

**Table 4:** Multivariate regression analysis for predicting factors of pCR rate

Variables		Adjusted OR (95% CI)	*P*
MEG3 rs10132552	CC + TC vs. TT	2.79(1.096–7.103)	0.031*
Tumor size	Continuous Variable	1.002(0.96–1.045)	0.943
Age	Continuous Variable	0.951(0.907–0.996)	0.035*
Ki67	Continuous Variable	1.059(1.033–1.086)	< 0.001*
Her2 expression	Positive vs. negative	11.718(3.974–34.554)	< 0.001*
Hormone receptor	Positive vs. negative	0.241(0.071–0.811)	0.022*

Abbreviations: *OR* odds ratio, *HER2* human epidermal growth factor receptor −2

*P < 0.05

### LncRNAs polymorphisms and prognosis

**Fig. 1:**
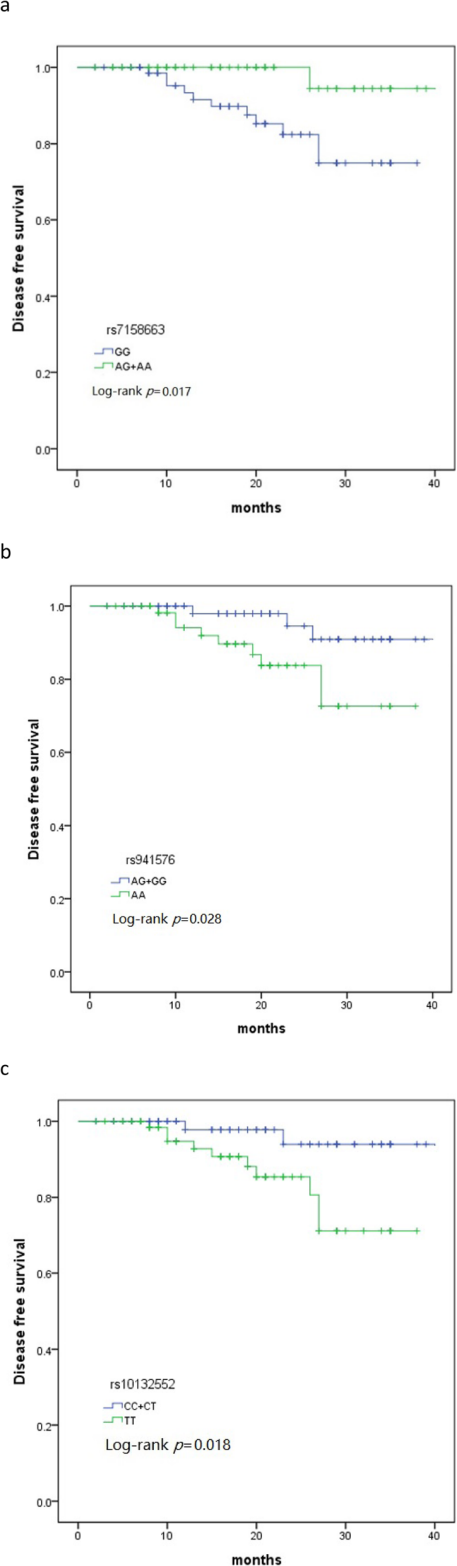
Kaplan-Meier Analysis of Disease-Free Survival. Disease-free survival by rs7158663 dominant model (**a**), rs941576 dominant model (**b**) and rs10132552 dominant model (**c**)

The median follow-up was 20 (2–40) months. The result showed that DFS in patients with MEG3 rs7158663 AG + AA genotype was better than that with GG genotype, and DFS was 94.4 and 85.3% (*p* = 0.017), respectively. In patients with rs941576 AG + GG genotype, the DFS was 98%, which was better than 89.7% (*P* = 0.028) in patients with AA genotype. The DFS of patients with rs10132552 CC + CT was 94%, which was significantly better than that with TT genotype (90.7%) (*P* = 0.018) (Fig. 1).

Linkage disequilibrium analysis indicated the SNPs rs10132552 and rs941576(r^2^ = 0.842, D’ = 0.987) were strongly linked. We further analyzed them as rs10132552 TT+ rs941576 AA haplotype which was significantly associated with poor DFS (HR = 0.257, 95% CI 0.069–0.951, *p* = 0.042) when it’s compared with other haplotypes. When considered with the rs7158663, patients with rs10132552TT+ rs941576AA + rs7158663GG were also significantly associated with poor DFS (HR = 0.175, 95% CI 0.047–0.648, *p* = 0.009). Multivariate analysis demonstrated the similar results (Table 5). In multiple stepwise selection Cox models, rs10132552 TC + CC (adjusted HR = 0.127, 95% CI 0.22–0.728, *p* = 0.02) and rs941576 AG + GG (adjusted HR = 0.183, 95% CI 0.041–0.807, *p* = 0.025) patients were also significantly associated with good DFS when adjusted by ki67, tumor size, lymph nodes, hormone receptor, HER2 expression and age.

**Table 5:** DFS according to MEG3 Polymorphisms

Gene	SNP	Genotype	DFS HR (95% CI)	*P*	DFS Adjusted HR (95% CI)	*P*
MEG3	rs10132552	TT	1		1	
		TC + CC	0.193(0.042–0.884)	0.034*	0.127(0.22–0.728)	0.02*
	rs941576	AA	1		1	
		AG + GG	0.257(0.069–0.951)	0.042*	0.183(0.041–0.807)	0.025*
	rs7158663	GG	1		1	
		AG + AA	0.124(0.016–0.964)	0.046*	0.155(0.019–1.236)	0.078
	rs10132552+ rs941576	TT + AA	1		1	
		others	0.257(0.069–0.951)	0.042*	0.183(0.041–0.807)	0.025*
	rs10132552+ rs941576 + rs7158663	TT + AA+GG	1		1	
		Others	0.175(0.047–0.648)	0.009*	0.116(0.025–0.552)	0.007*

Adjusted by ki67, tumor size, lymph nodes, hormone receptor, HER2 expression and age

Abbreviations: *HR* hazard ratio; DFS Disease-free survival, *HER2* human epidermal growth factor receptor −2

*P < 0.05

## Discussion

In this study, we detected the SNPs of long chain non-coding RNA MEG3 and analyzed the relationships between the polymorphisms and clinicopathological features, neoadjuvant chemotherapy sensitivity, prognosis and the toxicities of breast cancer patients. As far as we know, this is the first time to report the relationship between MEG3 lncRNA polymorphisms, efficacy and prognosis in locally advanced breast cancer patients who received neoadjuvant chemotherapy.

In our exploratory analysis, patients containing T allele in rs10132552 had higher level of ki67. MEG3 as a kind of tumor suppressor lncRNA, its mechanism of action has been widely studied in the occurrence and metastasis of tumor. Zhang’s study showed that MEG3 could reduce gliomas growth, tumor volume and the expression of ki67 [18]. Our result indicated that the MEG3 polymorphism was also associated with cell growth in breast cancers.

We observed that patients with MEG3 rs10132552 TC + CC genotype tended to achieve higher pCR rate than those with major allele homozygous. In Silico’s analysis, MEG3 rs10132552 was reported to change the structure of the transcript when the T allele was substituted by the C allele, and change the minimum free energy from − 150.6 kcal/mol to − 153.3 kcal/mol, which might alter the local RNA folding structure [19]. The change of structure might alter its potential function via certain regulating signals, resulting in different response to the therapy. In MEG3 overexpressing bladder cancer, cisplatin could significantly induce cell apoptosis, down-regulate bcl2 expression and up-regulate cleaved-caspase-3 and bax expression [20]. Wang’s study showed that nasopharyngeal carcinoma patients with MEG3 rs10132552 CT genotype had a better response to treatment (OR = 0.261, *p* = 0.015) [19]. In lung cancer, MEG3 could enhance the chemosensitivity through regulation the WNT/beta catenin signaling pathway and miR-21-5p/SOX7 axis [21, 22]. The regulative effect of MEG3 on miR-214 expression was associated with cisplatin resistance in ovarian cancer cells [23]. In breast caner cells, MEG3 inhibits cell growth and induces apoptosis, partially via the activation of the ER stress, nuclear factor κB (NF-κB) and p53 pathways, and that NF-κB signaling is required for MEG3-induced p53 activation in breast cancer cells [24]. These pathways might be the potential function for MEG3 to affect the response to chemotherapy in breast cancer patients.

We also observed that patients with MEG3 rs10132552 TT had worse DFS both in univariate and multivariate analysis. Perhaps this might be owing to the lower pCR rate of the patients with this genotype. MEG3 expression was reported to be an independent prognostic factor in breast cancer [25]. In other tumors, such as gastric cancer, overexpression of MEG3 could decrease the proliferation and metastasis via p53 signaling pathway [26]. MEG3 was also reported to suppress pancreatic neuroendocrine tumor growth by down regulating miR-183/BRI3 axis [27]. MEG3 could regulate the TGF-β pathway through formation of RNA-DNA triplex structures and finally target chromatin [28]. As a result, the patients with rs10132552 TT genotype had a substantially worse DFS than other cohorts. In addition, our data showed rs941576 which located in the intron of MEG3 was associated with DFS, too. There are few reports of this loci in tumors, and it was reported to be associated with fetal growth [29] and type I diabetes [30, 31]. Its effects on the survival of breast cancer patients might be associated with rs10132552.

The present study had some limitations. Our survival analyses were focused on DFS, the data of OS are not available now. Whether the MEG3 SNPs will be associated with the overall survival needs further study. The precise mechanisms of SNPs and efficacy remain unknown, and basic research is also necessary to study.

## Conclusions

In conclusion, MEG3 rs10132552 was associated with the cisplatin-containing chemotherapy response in breast cancer patients, and MEG3 rs10132552 and rs941576 were associated with disease free survival. All these SNPs might be considered as potential predictive markers for cisplatin-based neoadjuvant chemotherapy for breast cancer patients.

## Additional file

Additional file 1: **Table S1.** Detailed primer sequences of SNPs in MEG3 LncRNA. **Table S2.** Correlation between MEG3 rs941576 and rs7158663 and clinic-pathological parameters. **Figure S1.** Trial design (DOC 150 kb)

## Data Availability

The datasets used and/or analyzed during the current study are available from the corresponding author on reasonable request.

## Abbreviations

CI: confidential interval
DFS: disease-free survival
ER: estrogen receptor
HER2: human epidermal growth factor receptor − 2
HR: hazard ratio
lncRNA: Long non-coding RNA
MEG3: maternally expressed gene3
NF-κB: nuclear factor κB
OR: odds ratio
pCR: pathological complete remission
PR: progesterone receptor
SNPs: single nucleotide polymorphisms
