# Supplementary material for: Association of LncRNA MEG3 polymorphisms with efficacy of neoadjuvant chemotherapy in breast cancer
Source: BMC Cancer. 2019 Sep 5;19:877. doi: 10.1186/s12885-019-6077-3 (PMC6727505; doi:10.1186/s12885-019-6077-3)
Supplement: Supplementary file 1 — Table S1. Detailed primer sequences of SNPs in MEG3 LncRNA. Table S2. Correlation between MEG3 rs941576 and rs7158663 and clinic-pathological parameters. Figure S1. Trial design (DOC 150 kb) [file 12885_2019_6077_MOESM1_ESM.doc]

Figure S1 Trial design


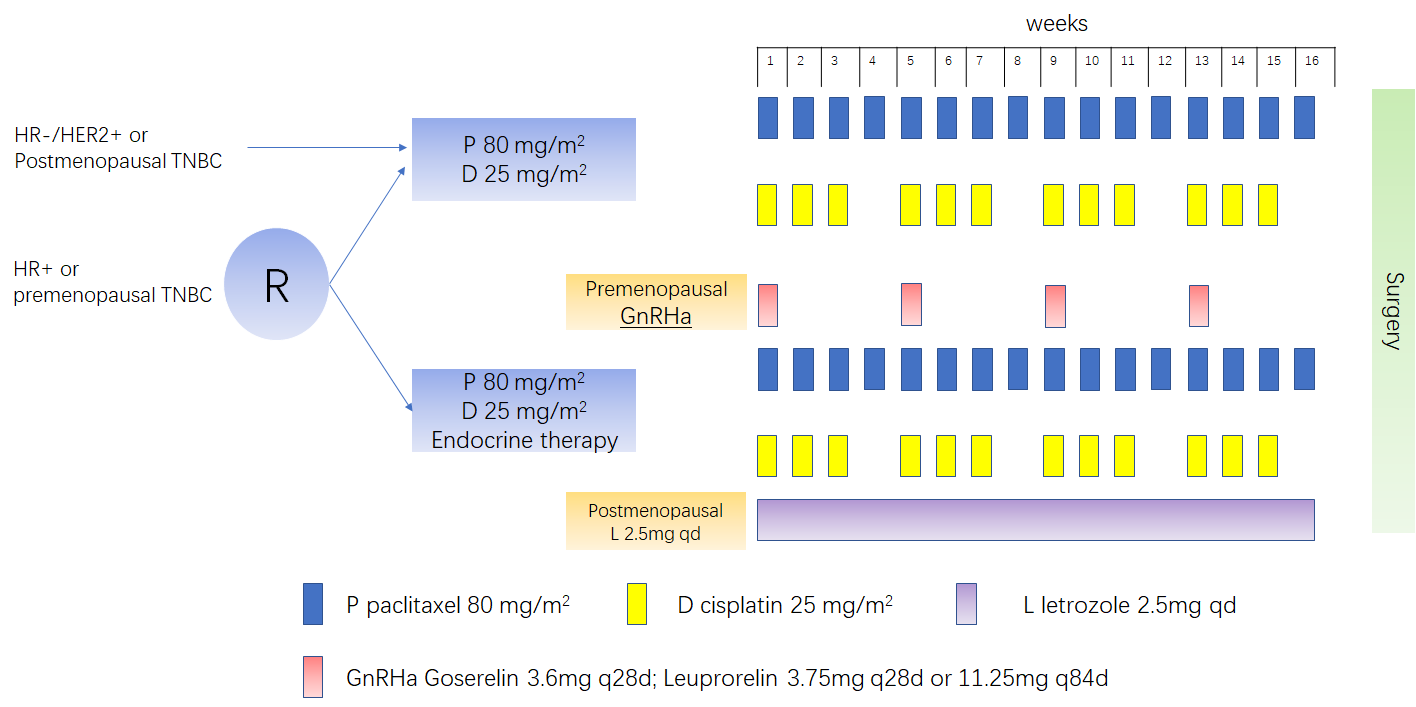


All patients enrolled in the trial were scheduled to receive paclitaxel, 80mg/m^2^ given once a week for 16weeks; and cisplatin 25 mg/m^2^ given weekly on days 1, 8 and 15 every 28 days for 4 cycles. Patients with hormone receptor-positive cancer or premenopausal patients with triple negative breast cancer were randomly assigned to receive endocrine therapy. Letrozole for postmenopausal women were given 2.5mg daily and gonadotropin releasing hormone agonist for premenopausal women were given every 28 days (goserelin 3.6mg or leuprorelin 3.75mg) or leuprorelin 11.25mg every 84 days.

Abbreviations: HR hormone receptor, GnRHa [gonadotropin-releasing hormone agonist](http://www.baidu.com/link?url=YZcSYok4W7FxNxcTF4_n6DRdYydAS7T-U7mCmH2e94qoN9lLwzZwCF_nvVfyVW0kQtAxGHvjdSlTyd2yylrEQ3FWK8R8dRSkR18l5yO4B7C), TNBC triple-negative breast cancer

Table S1. Detailed primer sequences of SNPs in MEG3 LncRNA

| rs Number | Primer sequence |
| --- | --- |
| rs10132552 |  |
| Primer 1 | ACGTTGGATGATACTCTAACTGAGCCCCAC |
| Primer 2 | ACGTTGGATGTTTGTCCCTCCCCAGTTCCT |
| rs941576 |  |
| Primer 1 | ACGTTGGATGTTGGGTCAGTGTTTCAGAGC |
| Primer 2 | ACGTTGGATGTGCAGTGTCTGCAGGGCTC |
| rs7158663 |  |
| Primer 1 | ACGTTGGATGTGAGATTCGGGATAGGGTTC |
| Primer 2 | ACGTTGGATGGTCTGGTACAGAAAGAACCG |

Table S2. Correlation between MEG3 rs941576 and rs7158663 and clinic-pathological parameters

|  | | rs941576 n(%) | | | P value | | | |
| --- | --- | --- | --- | --- | --- | --- | --- | --- |
|  |  | AA | AG | GG | Dominant | Recessive | co-dominant | Additive |
| T stage | 1~2 | 31(45.6) | 30(46.9) | 7(77.8) | 0.545 | 0.067 | 0.77 | 0.184 |
|  | 3~4 | 37(54.4) | 34(53.1) | 2(22.2) |  |  |  |  |
| Lymph node status | Negative | 6(9.2) | 12(19.4) | 2(22.2) | 0.085 | 0.51 | 0.161 | 0.22 |
|  | Positive | 59(90.8) | 50(80.6) | 7(77.8) |  |  |  |  |
| ER | Negative | 22(31.9) | 18(27.3) | 2(22.2) | 0.491 | 0.636 | 0.646 | 0.752 |
|  | Positive | 47(68.1) | 48(72.7) | 7(77.8) |  |  |  |  |
| PR | Negative | 15(21.7) | 14(21.2) | 1(11.1) | 0.797 | 0.458 | 0.918 | 0.757 |
|  | Positive | 54(78.3) | 52(78.8) | 8(88.9) |  |  |  |  |
| HER2 | Negative | 47(68.1) | 41(62.1) | 4(44.4) | 0.311 | 0.21 | 0.685 | 0.35 |
|  | Positive | 22(31.9) | 25(37.9) | 5(55.6) |  |  |  |  |
| Ki67 | Low expression | 16(24.6) | 13(21) | 4(44.4) | 0.927 | 0.144 | 0.412 | 0.306 |
|  | High expression | 49(75.4) | 49(78) | 5(55.6) |  |  |  |  |
| Menopausal status | Premenopausal | 30(43.5) | 29(43.9) | 3(33.3) | 0.922 | 0.543 | 0.844 | 0.83 |
|  | Postmenopausal | 39(56.5) | 37(56.1) | 6(66.7) |  |  |  |  |

|  | | rs7158663 n(%) | | | P value | | | |
| --- | --- | --- | --- | --- | --- | --- | --- | --- |
|  |  | GG | GA | AA | Dominant | Recessive | co-dominant | Additive |
| T stage | 1~2 | 38(47.5) | 25(47.2) | 5(62.5) | 0.843 | 0.405 | 0.845 | 0.707 |
|  | 3~4 | 42(52.5) | 28(52.8) | 3(37.5) |  |  |  |  |
| Lymph node status | Negative | 9(11.7) | 10(19.6) | 1(12.5) | 0.256 | 0.856 | 0.211 | 0.457 |
|  | Positive | 68(88.3) | 41(80.4) | 7(87.5) |  |  |  |  |
| ER | Negative | 26(31.7) | 12(22.2) | 4(50) | 0.44 | 0.182 | 0.156 | 0.202 |
|  | Positive | 56(68.3) | 42(77.8) | 4(50) |  |  |  |  |
| PR | Negative | 17(20.7) | 10(18.5) | 3(37.5) | 0.972 | 0.232 | 0.596 | 0.467 |
|  | Positive | 65(79.3) | 44(81.5) | 5(62.5) |  |  |  |  |
| HER2 | Negative | 53(64.6) | 36(66.7) | 3(37.5) | 0.83 | 0.11 | 0.591 | 0.27 |
|  | Positive | 29(35.4) | 18(33.3) | 5(62.5) |  |  |  |  |
| Ki67 | Low expression | 19(24.1) | 14(28) | 0(0) | 0.945 | 0.124 | 0.438 | 0.269 |
|  | High expression | 60(75.9) | 36(72) | 7(100) |  |  |  |  |
| Menopausal status | Premenopausal | 37(45.1) | 21(38.9) | 4(50) | 0.565 | 0.683 | 0.434 | 0.711 |
|  | Postmenopausal | 45(54.9) | 33(61.1) | 4(50) |  |  |  |  |

Abbreviations: ER estrogen receptor; PR progestogen receptor; HER2 human epidermal growth factor receptor -2
